# Editorial: Soybean molecular breeding and genetics

**DOI:** 10.3389/fpls.2023.1157632

**Published:** 2023-02-28

**Authors:** Guo-Liang Jiang, Istvan Rajcan, Yuan-Ming Zhang, Tianfu Han, Rouf Mian

**Affiliations:** ^1^ Agricultural Research Station, Virginia State University, VA, Petersburg, United States; ^2^ Department of Plant Agriculture, Ontario Agriculture College, University of Guelph, Guelph, ON, Canada; ^3^ Crop Information Center, College of Plant Science and Technology, Huazhong Agricultural University, Wuhan, China; ^4^ Institute of Crop Sciences, Chinese Academy of Agricultural Sciences, Beijing, China; ^5^ Soybean and Nitrogen Fixation Unit, United States Department of Agriculture-Agricultural Research Service (USDA-ARS), Raleigh, NC, United States

**Keywords:** *Glycine max*, breeding and genetics, genomics, QTL, GWAS, genomic selection

Soybean [*Glycine max* (L.) Merr.] is one of the most important supplies of plant protein and oil for human consumption as well as a major source of protein meal for animal feeds. Both the progress in technology and the increasing market demand have driven a sustainable increase of the world soybean crop both in acreage and yield since 1990s, especially in North and South America. On the other hand, soybean production still faces many challenges, such as the impacts of climate changes and environmental stresses, damages caused by diseases and pests, and demand of higher yield potential and improved quality for diverse end uses. Developing new cultivars with desired characteristics through genetic improvement is a key to the solution to these challenges. Molecular breeding integrated with novel technologies greatly helps the realization of the goal. This Research Topic provides a platform to present research progress and discuss potential approaches to facilitate further development. Twenty-four articles published involve a wide range of areas, mainly addressing important traits and breeding strategies, such as resistance to diseases, agronomic and yield traits, and nutritional profiles. In addition to the identification of quantitative trait loci (QTL) and candidate genes associated with target traits, molecular mechanisms underlying traits are also explored.

## Resistance to diseases

Growing resistant cultivars is the most economically effective method of preventing yield loss, and resistance to diseases and/or pests is always one of the most important breeding objectives in soybean. Soybean cyst nematode (SCN, *Heterodera glycines* Ichinohe) is a devastating pathogen for soybean production worldwide (Wei et al.). Using a population of 392 F_2:8_ recombinant inbred lines (RILs) derived from the cross Zheng 9525 × Handou 10, Wei et al. identified seven QTL with additive effects. Among these QTL, three on Chromosomes 7, 8, and 18 exhibited resistance to two races, SCN HG type 2.5.7 (race 1) and 1.2.5.7 (race 2). They assumed that the previously reported QTL on chromosomes 8 and 18 were most likely overlapped with *rhg1* and *Rhg4* loci, respectively, while the QTL on chromosome 7 was novel. Using an association panel of 183 representative soybean accessions, Sun et al. evaluated the resistance to soybean frogeye leaf spot (FLS) race 1 and performed genome-wide association study (GWAS) to identify quantitative trait nucleotides (QTNs) and the candidate genes. Thirteen novel marker-resistance association signals were identified, and 19 candidate genes were found within the 200-kb flanking regions of these 13 peak SNPs. They preliminarily proved that four genes played important roles in soybean resistance to FLS race 1.

Phytophthora root and stem rot caused by the soil-borne oomycete *Phytophthora sojae* is a yield-limiting soybean disease (Robertson et al., 2009; Karhoff et al.). A major quantitative disease resistance locus on chromosome 18, referred to as *QDRL-1*8, explains up to 45% of the phenotypic variation (Lee et al., 2014). By high-resolution mapping, Karhoff et al. narrowed the *QDRL* interval down to 3.1 cM (or 731 kb), which contains 82 predicted genes. The resistant allele of *QDRL-18* was not shown to have evidence of yield drag in the fields lacking disease pressure, but significantly increased yield under disease conditions. To reveal novel regions of soybean genome associated with resistance to southern root-knot nematode, Vieira et al. conducted a machine learning based GWAS in a population of 717 breeding lines derived from 330 bi-parental populations, using random forest and support vector machine algorithms. They compared the powers of different GWAS methods/models in detecting minor effect loci.

## Seed composition and nutritional profiles

Seed composition and nutritional profiles play an important role in the quality and market prices of soybean. Low concentration of methionine, an essential amino acid, limits the nutritional utility of soybean protein. Singer et al. performed a GWAS using 311 soybean accessions selected from maturity groups (MGs) IV and V, which were genotyped with a total of 35,570 SNPs. Across four environments, 23 new SNPs were identified to be associated with methionine content. The strongest associations were found on chromosomes 3, 8 and 16. They also suggested that genomic selection utilizing a significant subset of SNPs (He et al., 2019) might be a practical tool for improving methionine content. The nutritional value of soybean oil is determined by seed fatty acid composition, especially the relative contents of three unsaturated fatty acids (Ensminger and Ensminger, 1993). To identify the candidate genes and related pathways involved in the regulation of unsaturated fatty acids during seed development in soybean, Liu et al. selected two soybean lines from 314 cultivars and landraces originated in southern China, which were different in unsaturated fatty acid contents, and performed RNA-seq analysis in soybean seeds at three developmental stages. They identified a series of genes and pathways related to fatty acid metabolism, and found that six genes in functions were highly associated with the contents of oleic and linoleic acid.

The introgression of alleles from wild soybean (*Glycine soja* Siebold and Zucc.) is a worthwhile option to enhance genetic diversity and germplasm for traits of value (Yang et al.). To examine the genetic architecture responsible for seed protein and oil, Yang et al. conducted linkage mapping using a RIL population derived from a cross of *G. max* cultivar ‘Osage’ and *G. soja* accession PI 593983. They identified seven significant QTL on chromosomes 14 and 20 for seed protein and on chromosome 8 for seed oil. Within the significantly associated genomic regions identified, eight genes were considered as candidate genes. Through a restricted two-stage multi-locus GWAS with 15,501 SNP linkage-disequilibrium block markers in a population of 361 germplasm accessions from Northeast China, Feng et al. identified 73 QTL associated with seed protein, explaining 71.70% of phenotypic variation, of which 28 were new ones. They also annotated a total of 120 candidate genes and functionally classified into 13 categories. A group of Canadian scientists have dedicated a collaborative effort to soybean improvement, including GWAS of multiple traits, to identify novel alleles underlying seed yield and seed quality and agronomic traits coming from modern Chinese parents (Priyanatha et al.). They have developed a genomic panel consisting of modern Canadian (CD) and Chinese (CH) cultivars, and the progenies of CD × CH crosses, field-tested it and subjected it to genotyping with 32K high-quality SNPs developed through genotyping-by-sequencing. A putative gene has been identified for each of the seed yield and seed oil and protein concentration (Priyanatha et al.).

Isoflavone is an important secondary metabolite in soybean and is beneficial to human health. Using a RIL population derived from ‘Zhongdou27’ and ‘Hefeng25’ and a high density linkage map based on whole-genome resequencing, Chen et al. identified 41 QTL associated with quality traits. Of these QTL, 27 were for isoflavones, seven for protein, four for oil, and three related to both protein and oil. Li et al. identified 15 QTL associated with isoflavone content, using a mapping population of 119 F_5:18_ RILs, developed by crossing soybean cultivar “Zhongdou27” with “Dongnong8004”. A novel locus, *qISO19-1*, was fine-mapped to a 62.8 kb region on chromosome 19 using a BC_2_F_2_ population. They also considered *GmMT1* as a candidate gene for this locus and confirmed it by overexpression in Arabidopsis and soybean cultivars.

## Agronomic and yield traits

Drought causes significant soybean yield losses each year in rain-fed production systems in the world (Chamarthi et al.). Genetic improvement of soybean for drought tolerance is a cost-effective approach to stabilize yield under rain-fed management. By association mapping in a panel of 200 diverse MG IV accessions using 34,680 SNPs, Chamarthi et al. identified 188 significant SNPs associated with canopy wilting, with 152 loci tagged. Of these SNPs, 87 were coincident with those previously reported that likely tagged 68 loci, and 101 were novel ones that likely tagged 84 loci. In addition, 183 candidate genes for both coincident SNPs and novel SNPs were identified in the vicinity of those significant SNPs, and among these genes, 57 SNPs were present within genes coding for proteins with biological functions involved in plant stress responses.

Plant height is important for soybean breeding since it is closely related to plant shape and yield. Based on a high-density genetic linkage map constructed in a RIL population derived from Dongnong L13 × Henong 60, Wang et al. identified 33 QTL associated with plant height, of which five were repeatedly detected in multiple environments. In addition, a total of 62 plant height QTNs were detected, of which 26 were detected repeatedly under multiple methods through multi-locus association analysis in a population of 455 accessions genotyped with 63,306 SNP markers. Two candidate genes associated with plant height, *Glyma.02G133000* and *Glyma.05G240600*, were predicted and validated (Wang et al.). Using fixed and random model circulating probability unification (FarmCPU) method, Priyanatha et al. also performed GWAS for plant height, days to maturity, lodging score, 100 seed weight, and seed yield. Their study provides insight into potentially valuable genetic resources residing in Chinese modern cultivars that breeders may use to further improve soybean seed yield and seed quality traits.

Seed number per pod (SNPP) is an important yield component and a practicable target to perform direct selection in field breeding trials. However, progress in soybean breeding for this trait is limited, while many other legumes have much larger SNPP than soybean, for instance cowpea has 12 SNPP on average (Liu et al.). To explore possible molecular mechanisms for the SNPP difference in soybean and cowpea, Liu et al. attempted to identify *PIN1* and *CKX* gene families that regulate SNPP in Arabidopsis, analyzed the differences of auxin and cytokinin pathways. They constructed interaction networks on *PIN1*, *CKX* and yield related genes in soybean and cowpea, and detected their network differences in the two legumes. Main stem node number (MSNN) of soybean is another important yield-related trait. Li et al. used 144 four-way RILs to identify QTL for MSNN at two densities of plants/ha in five environments by linkage and association studies. As a result, 40 and 28 QTL were identified for the two densities, respectively. In addition, they predicted four candidate genes associated with MSNN.

Early leaf senescence phenotype in soybean could be helpful to partition dry matter to seeds, shorten the maturation period and prevent green stem disorder. From a high-density mutation library, Yamatani et al. identified two early leaf senescence soybean mutant lines, els1-1 (early leaf senescence 1) and els1-2. The chlorophyll contents of both els1-1 and els1-2 were low in pre-senescent leaves, and degraded rapidly in senescent leaves. It was indicated that the ELS1 was involved in chlorophyll biosynthesis during leaf development and chlorophyll degradation during leaf senescence. On the other hand, degradation of chlorophyll in mature soybean seeds is closely related to the development of their yellow color (Tokumitsu et al.). Tokumitsu et al. examined G and its homologue G-like, and their mutant alleles, and investigated the relationship between these genes and chlorophyll accumulation in the seed coats of mature seeds. Their study suggested that high expression levels of G would result in chlorophyll accumulation that exceeded its metabolism in the seeds of yellow soybean, and the mutation of the G locus alone could be essential for establishing yellow seed coat of soybeans, which is the major type of modern soybean cultivars.

Soybean pubescence plays an important role in insect resistance, and tolerance to drought and other stresses (Li et al.). By QTL mapping of pubescence traits using a high-density inter-specific linkage map of a RIL population, Li et al. observed that pubescence length (PL) was negatively correlated with pubescence density (PD). They identified ten QTL for PL and nine for PD on six and five chromosomes, respectively, which explained 3.0–9.9% and 0.8–15.8% of phenotypic variance. They also identified 21 and 12 candidate genes related to PL and PD.

## Breeding methodologies and approaches

To facilitate breeding progress and enhance breeding efficiency, scientists have explored breeding methodologies and approaches integrated with new technologies. Genomic selection and marker-assisted recurrent selection have been applied to improve quantitative traits in cross-pollinated species (Massman et al., 2013; Beyene et al., 2015; Beyene et al., 2016). However, such a selection scheme is not practicable in self-pollinated crops because of laborious crossing (Bernardo, 2010). Sekine et al. developed a simulation-based selection strategy that uses a trait prediction model based on genomic information to predict the phenotype of the progeny for all possible cross combinations. These predictions may be used to select the best cross combinations for the improvement of given traits. Shook et al. presented the approach “Parental Allele Tracing, Recombination Identification, and Optimal predicTion (PATRIOT)” that uses marker data to allow for a rapid identification of lines carrying specific alleles, increases the accuracy of genomic relatedness and diversity estimates, and improves genomic prediction (GP). Leveraging identity-by-descent relationships, PATRIOT improved the GP accuracy by 16.6% relative to the traditional rrBLUP method. Zhou et al. reported an improved whole-genome sequencing-based bulked segregant analysis method, termed as M2-seq, which was regarded as an efficient mutant gene mapping tool, comparable to the previously reported approaches, such as Mutmap and Mutmap+ that require studying M3 or advanced selfed generations.

For molecular breeding, agronomic traits, such as growth habit, stress tolerance, seed color and yield, have been the targets. When choosing elite germplasm with beneficial alleles, however, it should be taken into consideration that the genes governing these traits often undergo posttranscriptional modifications (Ku et al.). The omics approaches allow the scientific community to successfully identify genomic regions associated with traits of interest for marker-assisted breeding (Ku et al.). Ku et al. presented a review of related research works and discussed the posttranscriptional modifications of genes related to desirable agronomic traits in soybean and other crops.

Collaboration is an important feature and a key to success for today’s plant breeding. Belzile et al. presented a collaborative project named as the SoyaGen project involving Canadian soybean researchers and breeders from a nation-wide and international community. It aims to develop genomics-derived solutions to real-world challenges faced by breeders. Based on the needs of the stakeholders, the research efforts were focused on maximizing realized yield through optimization of maturity and improved disease resistance. Multi-parent advanced generation inter-cross (MAGIC) populations derived from breeder-relevant germplasm provide platforms for increased recombination and high-resolution or fine mapping of quantitative traits in crop species (Cavanagh et al., 2008). To introduce soybean MAGIC population as an unprecedented platform for genotypic and phenotypic investigation of agronomic and seed quality traits in soybean, Hashemi et al. have established an eight-parent MAGIC population, comprising 721 RILs, of which the parents were genetically diverse elite cultivars carrying different agronomic and seed composition characteristics. The SoyMAGIC population is expected to accelerate further genomic studies and the development of soybean cultivars with improved seed quality traits through the development and implementation of reliable molecular-based toolkits.

In summary, the studies presented in this Research Topic will help to improve the understanding of important traits and their molecular mechanisms, and develop effective and efficient approaches to trait improvement. They will be beneficial for future work on QTL mapping, marker-assisted selection, map-based cloning, GWAS, genomic selection, gene editing, and breeding by design in soybean. It is anticipated that soybean molecular breeding will be more extensively developed, from laboratory research to field selection, and new cultivars with desirable traits will be developed through such integrated approach.

## Author contributions

G-LJ wrote the manuscript. All authors reviewed, edited and approved the manuscript.
